# High-Precision Field- Effect Transistor Biosensor for Analyzing Differential Effects of Anti-Cancer Drugs on Cancerous and Non-Cancerous Cells

**DOI:** 10.3390/bios15020125

**Published:** 2025-02-19

**Authors:** Can Li, Can Hu, Ling Li, Feng He, Xiaofei Li

**Affiliations:** 1 Engineering Research Center of TCM Intelligence Health Service, School of Artificial Intelligence and Information Technology, Nanjing University of Chinese Medicine, Nanjing 210023, China; 2 School of Computer and Software, Hohai University, Nanjing 211100, China; 3 College of Computer and Information Engineering (College of Artificial Intelligence), Nanjing Tech University, Nanjing 211816, China; 4 Department of Sports Medicine, The First People’s Hospital of Lianyungang, Affiliated Lianyungang Hospital of Xuzhou Medical University, The First Affiliated Hospital of Kangda College of Nanjing Medical University, Lianyungang 222599, China; 5 Department of Sports Medicine and Adult Reconstructive Surgery, Drum Tower Clinical Medical College of Nanjing Medical University, Nanjing 210008, China

**Keywords:** field-effect transistor biosensors, rapid detection, anti-cancer drugs, camptothecin, doxorubicin, drug action speed

## Abstract

A high-precision biosensor technique is introduced, offering the capability to independently evaluate the effects of anti-cancer drugs on both cancerous (RAJI) and non-cancerous (WIL2S) cells. By analyzing and fitting current change curves and transfer characteristic curves under two drugs, camptothecin and doxorubicin, this technique quantifies both the magnitude of drug-induced current changes in cells and the rate of drug entry into cells. Flow cytometry was utilized to validate the entry rates of two drugs, camptothecin and doxorubicin, into the cells. The biosensor leverages the exceptional sensitivity of two-dimensional electron gas to detect proximal charge variations at ultralow concentrations, even in fluids with high ionic strength. The findings reveal that anti-cancer drugs have a more pronounced impact on tumor cells, with the effects and interaction speeds differing across normal cells and tumor cells. This innovative approach not only enhances our understanding of the specificity and action mechanisms of anti-cancer drugs but also provides a valuable tool for screening potential tumor anti-cancer drugs and advancing targeted cancer therapies.

## 1. Introduction

Malignant tumors pose a significant threat to human health, with drug therapy being one of the most common and essential treatment approaches [1,2]. Recently, precision medicine has emerged as a leading trend in cancer treatment [3,4,5]. To achieve effective and targeted drug therapy for malignant tumors, drug screening plays a crucial role [6]. Anti-cancer drugs exert cytotoxic effects on neoplastic cells, but the impact of different drugs on the same cell type can vary significantly; also, the strong killing effect of anti-tumor drugs on normal tissues may limit their suitability for tumor treatment [7,8]. The permeability of the cell membranes is regulated by the opening and closing of membrane channels, which control the flow of ions in and out of the cell, thus influencing the cell’s functional state. During drug therapy, drug molecules affect the activity of membrane channels, altering the balance of ion concentrations inside and outside the cell, which in turn changes the cell’s metabolism and signaling processes [9,10]. Drug–membrane interactions can induce changes in the physical properties of the cell membrane, reflecting the drug’s activity within the membrane, which in turn affects the physiological functions of cells [11]. Specifically, drug molecules can alter the physical and chemical properties of the membrane, such as its fluidity, thickness, and surface charge, by binding to membrane lipids and membrane proteins [12]. These changes not only affect the stability of cell membranes but can also further regulate the entry and exit of ions and small molecules by altering the open and closed states of membrane channels, impacting cell metabolism, signal transduction, and other critical functions [13]. Therefore, examining the charge characteristics of cell membranes is vital for analyzing functional alterations in tumor cells, offering valuable insights for tumor detection and treatment. Investigating membrane charge alterations during the action of anti-cancer drugs is essential for the advancement of cancer therapy.

In electrophysiological research, cell electrical signals are typically recorded using patch clamp techniques [14]. However, patch clamping presents several challenges, including potential damage to the cell membrane [15], and it is limited to recording the excitability of individual cells. Additionally, the recording duration is often short, which restricts more complex analyses [16]. The electrochemical impedance spectroscopy [17] technique is a powerful technique for measuring the impedance of cells or tissues to an alternating current, enabling the inference of electrical and charge properties of the cell membrane. However, analyzing the resulting impedance spectra is inherently complex, requiring a nuanced interpretation of the data. This method also relies heavily on specialized equipment and technical expertise, with the associated instruments being relatively costly. Voltage-sensitive fluorescent dyes represent a vital tool for investigating signal transduction in neurons and other excitable cells [18]. By labeling the cell membrane with these dyes, researchers can monitor dynamic changes in membrane potential in real time [19]. Nevertheless, these dyes can exhibit cytotoxicity, which may interfere with experimental outcomes. Furthermore, the sensitivity of detection is strongly influenced by the choice of dye and the optimization of experimental conditions. Optogenetics offers a sophisticated approach for controlling cellular electrical activity by using light to activate specific photosensitive ion channels or proteins [20]. Real-time monitoring is achieved through electrophysiological recording techniques. However, the approach demands the precise genetic modification of cells through genetic engineering, making it technically challenging. Additionally, the requirement for specialized light sources and carefully controlled illumination parameters adds further complexity to its application.

AlGaN/GaN heterojunction field-effect transistors (AlGaN/GaN HFETs) offer high sheet carrier concentrations at the AlGaN/GaN interface, high saturation velocity, and a high breakdown field [21,22]. These properties make them suitable for high-temperature, high-power, high-frequency, and low-noise applications [23]. AlGaN/GaN HFETs are ion-impermeable and highly stable in electrolytes, making them ideal for detecting ultralow concentrations in fluids with high ionic strengths. The AlGaN/GaN heterojunction generates a high-conductivity region known as the two-dimensional electron gas (2DEG). The source-drain current in the 2DEG is highly sensitive to proximal charges; positive charges attract electrons, increasing the current flowing through drain and source (I_ds_), while negative charges repel electrons, decreasing I_ds_. Due to their superior performance, AlGaN/GaN HFETs have been widely used in applications such as DNA hybridization detection [24], cellular electrophysiological measurements [25], label-free detection of biological activity [26], extracellular signal recording, and ion detection [27].

In this study, a practical physical approach for the rapid and simple screening of drugs on cancerous and normal cells using AlGaN/GaN field-effect transistors (HEFTs) [24] has been established. This method leverages the high conductivity region of the AlGaN/GaN HFET to detect charge generation induced by drugs on B lymphoma cells and B lymphocytes. The GaN field-effect transistor was employed to evaluate the effects of two anti-cancer drugs, Camptothecin (CPT) and Doxorubicin (DOX), on different cell lines, specially RAJI and WIL2S cells. The results allowed for the analysis of drug effects and their rates of action on the cells. The study revealed that CPT significantly increased by 1.5 µA in RAJI cell fluid, while DOX showed minimal change in the same experimental conditions. Additionally, the action rates of CPT and DOX on different cell types were further analyzed and confirmed though flow cytometry.

## 2. Materials and Methods

### 2.1. AlGaN/GaN HFET Biosensor Preparation

**Figure 1:**
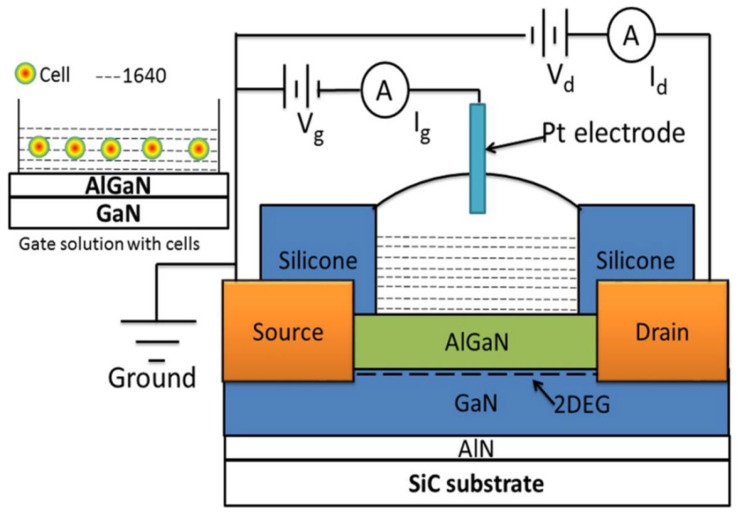
The cross-sectional view and device configuration of AlGaN/GaN HFET biosensors with RPMI 1640 culture medium addition to the gate area.

**Figure 2:**
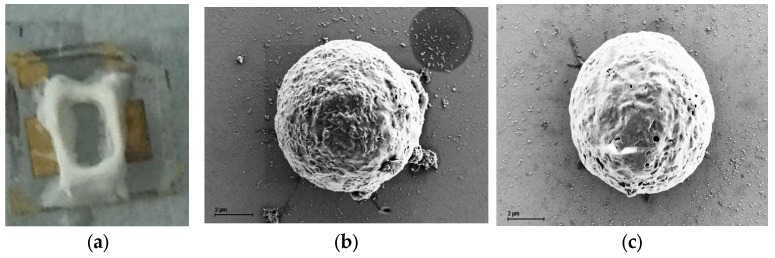
The device’s physical image and cell characterization image. (**a**) The picture of the AlGaN/GaN HFET biosensors. (**b**) A SEM image of a RAJI cell in the AlGaN/GaN HFET sensor in a concentration of about 5 cells/μL. (**c**) A SEM image of a WIL2S cell in the AlGaN/GaN HFET sensor in a concentration of about 5 cells/μL.

Before each use, the device was cleaned with deionized (DI) water and subsequently dried with nitrogen. Device characteristics were evaluated using a three-terminal configuration, with an inert metal-platinum (Pt) electrode serving as the gate to minimize the impact of measurement electrodes on the results (see Figure 1). A 2 μL volume of RPMI 1640 culture medium or cell fluid was applied to the fluid reservoir. A Pt reference electrode was positioned in the RPMI 1640 culture medium with its relative height set at 2 mm from the AlGaN surface using a precisely controlled XYZ probe arm. Figure 1 illustrates the basic connection mode, with the inset in the top-left corner showing cells suspended in solution on the AlGaN surface. Figure 2 presents the device’s physical image (Figure 2a) and cell characterization image by SEM (Figure 2b,c).

**Figure 3:**
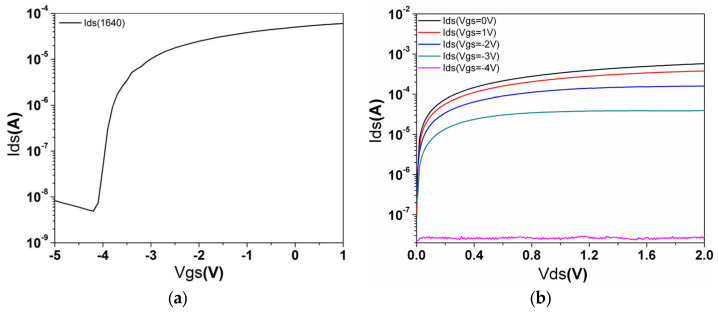
The electrical characteristics curves of the device. (**a**) $I_{ds}-V_{gs}$ characteristics of the AlGaN/GaN HFET sensor in 2 µL RPMI 1640 culture medium with an external bias to the gate from −5 V to 1 V. $V_{ds}$ is set as 0.1 V. (**b**) The $I_{ds}-V_{ds}$ curves of the AlGaN/GaN HFET sensor in RPMI 1640 culture medium at different $V_{gs}$ values in which $V_{ds}$ varies from 0 V to 2 V with an interval of 0.1 V.

Changes in the electrical characteristics were recorded using current–voltage measurement mode with a Semiconductor Parameter Analyzer (Keithly 4200SCS, manufactured by Keithley Instruments, which is headquartered in Cleveland, OH, USA). The measurements were taken at a drain-source voltage ($V_{ds}$) of 0.1 V, while the gate-source voltage ($V_{gs}$) varied from −5 V to 1 V in 0.1 V intervals (see Figure 3a). From the test data, the threshold voltage was determined to be $V_{gs}=-4.19V$ when using the RPMI 1640 culture medium. Figure 3b shows the $V_{ds}-I_{ds}$ curves when $V_{gs}$ ranges from −5 V to 0 V in 1 V intervals. The results indicate that the device operates normally.

### 2.2. Cell Lines and Reagents Preparation

WIL2S cells are B lymphocytes, while RAJI cells are B lymphoma cell lines. They were both purchased from ATCC. CPT was acquired from KeyGEN Biotech (also known as Jiangsu KeyGEN Biotech Co., Ltd., which is located at Nanjing, China) (Cat. NO: KGA8151), and DOX was obtained from Beijing Huafeng United Technology Co., Ltd., Beijing, China. Measurements were conducted using a Keithley 4200 SCS. Before conducting cell experiments, the cell fluid was processed by multiple centrifugations and supernatant removal to eliminate the influence of fetal bovine serum (FBS). The cell concentration for the experiments was adjusted to approximately 5 cells/μL. This concentration was achieved by first counting the cell concentration in the original cell fluid using a cell counter and then diluting or concentrating the solution as needed.

### 2.3. Experimental Setup and Drug Treatment Procedure for Sensor Response Experiments

**Figure 4:**
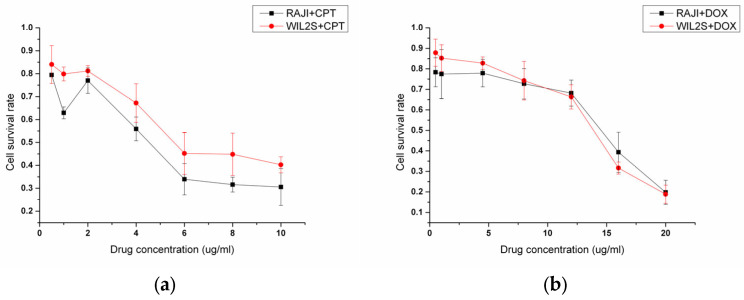
Drug concentration experiment results. (**a**) Cell survival curves following treatment with varying concentrations of CPT. (**b**) Cell survival curves following treatment with varying concentrations of DOX.

**Figure 5:**
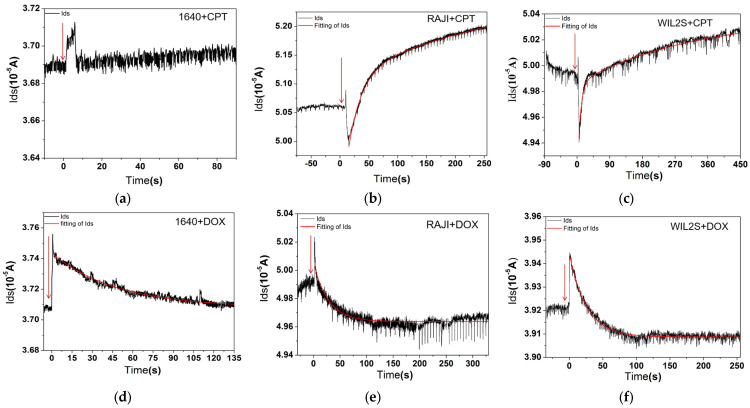
Current curves of source and drain vary over time. The current change of $I_{ds}$ with the addition of 2 μg/mL CPT to RPMI1640 culture medium (**a**), RAJI cell fluid (**b**), and WIL2S cell fluid (**c**), respectively. The current change of $I_{ds}$ after the addition of 10 μg/mL DOX to RPMI 1640 culture medium (**d**), RAJI cell fluid (**e**), and WIL2S cell fluid (**f**), respectively. $I_{ds}$ represents the current flowing through the drain and source electrodes. The red solid line in each graph indicates the fitting curve (fitting formula see Formula (1)) of $I_{ds}$ after drug addition. For the time axis, 0 s represents the time of drug addition.

**Figure 6:**
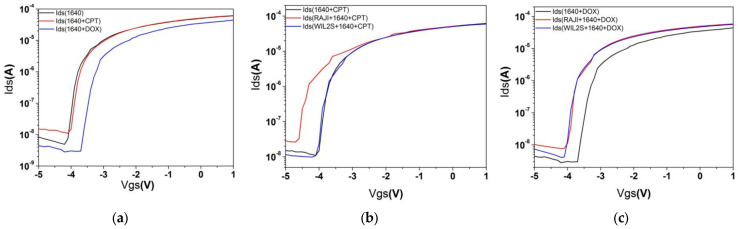
Transfer characteristic curves of the AlGaN/GaN HFET sensor under various conditions corresponding to Figure 5. (**a**–**c**) $V_{gs}{-I}_{ds}$ under different conditions, from which their threshold voltages ($V_{th})$ can be obtained by nonlinear fitting of the curves of the fast-growing part. The values are listed in Table 2. For the pure RPMI 1640 culture medium, the $V_{th}$ value is −4.19 V, which is not included in the table. Notes:$\;V_{gs}$ is the gate-source voltage of the sensor and $I_{ds}$ is the current flowing through the drain and source electrodes.

For the experiments, the device was cleaned with DI (deionized water) water and then dried by nitrogen. It was then fixed in the Semiconductor Parameter Analyzer 4200SCS. The source voltage was set as 0 V, drain as 0.1 V, and the gate voltage as 0 V for excluding the stimulus of gate voltage. Before conducting drug treatment experiments, we establish a drug concentration gradient, assess cell viability using the MTT assay, and select the highest drug concentration that maintains cell viability above 70%. A low concentration solution of 2 μg/mL CPT (see Figure 4a) or 10 μg/mL DOX (see Figure 4b) was prepared in dimethyl sulfoxide (DMSO). Once stabilized, 2 μL of the drug solution was added to the gate area and was continuously recorded throughout the procedure. The results are presented in Figure 5a–f. Figure 6a–c show the transfer characteristics under different conditions after drug treatment, used to assess changes in the threshold voltage.

### 2.4. Flow Cytometry Experiment

Both CPT and DOX are fluorescence compounds [28]. Therefore, the drug levels within cells at various time points can be detected using flow cytometry. CPT exhibits strong blue fluorescence when excited by ultraviolet light, with an excitation and emission of λ_ex_ = 370 nm and λ_em_ = 420 nm, respectively [29]. DOX has an absorption peak around 480 nm, and its fluorescence is detected at 560 nm [30,31]. Intracellular drug levels at different time points were measured using flow cytometry (Beckman, Model: Gallios, manufactured by Beckman Coulter, which is headquartered in Folsom, CA, USA), providing an alternative method to assess the action speed of drugs on cells.

### 2.5. Data Handling

Data from the AlGaN/GaN HFET biosensor tests, conducted using a Semiconductor Parameter Analyzer (Keithly 4200SCS), were acquired, processed, and presented using ORINGIN Pro 9.0 (Oringinlab Corporation, Northampton, MA, USA). The fitting formula of Figure 5 is

(1) $$I_{ds}=a\times e^{-\frac{t}{\tau_{1}}}+b\times e^{-\frac{t}{\tau_{2}}}+c$$

where τ represents the constant of the time process for transition reactions. It refers to the time required for a physical quantity to decay from its maximum value to 1/e of its maximum value. For a quantity that decays exponentially, the time required for the amplitude to decay to 1/e of its initial value is known as the time constant. Statistical analysis of the flow cytometry experiments was performed with quantitative data expressed as means and standard deviations (SDs). Two-tailed Student’s *t* tests were used to determine *p*-values, with statistical significance defined as *p* < 0.05. All experiments were conducted independently in triplicate.

## 3. Results

### 3.1. Device Performance of AlGaN/GaN HFET Biosensor

From the test data, the threshold voltage was determined to be $V_{gs}=-4.19V$ (see Figure 3a) when the gate fluid was RPMI 1640 culture medium. The data presented in Figure 3b show that I_ds_ increases with increasing V_gs_ at a constant V_ds_. Both Figure 3a,b indicate that the device is functioning normally.

### 3.2. I_ds_ Change in Drugs Action on Cells

The effects of CPT and DOX on cells are illustrated in Figure 5a–f. The addition of CPT to a RAJI cell fluid resulted in a significant increase in $I_{ds}$ (about 1.5 µA) after 250 s (Figure 5b). To rule out potential interactions between RPMI 1640 culture medium and CPT, rather than the drug’s effect on the cells, a control experiment was conducted with only RPMI 1640 culture medium and CPT, without cells. This control did not show a significant change in $I_{ds}$ when CPT was added (Figure 5a).

In comparison, the addition of CPT to WILS2 cells (Figure 5c) produced a less pronounced effect, if any, compared to the RAJI cells. A similar experiment was performed for DOX on RAJI cells (Figure 5e) and in a clean RPMI 1640 culture medium to exclude unexpected effects (Figure 5d). The results for DOX showed a slight decrease of $I_{ds}$ over time in all case involving cells, while the control experiment with RPMI 1640 culture medium alone did not exhibit notable effects (Figure 5f). The response time constant τ of different conditions corresponding to Figure 5a–f is listed in Table 1.

### 3.3. Transfer Characteristics of Different Conditions After Drug Treatment and Analysis of Charge of the Two Drugs

Figure 6a–c examine the transfer characteristics under different conditions after drug treatment to assess changes in the threshold voltage by comparing the threshold voltage values of the gate area upon adding CPT and DOX to the RPMI 1640 culture medium (as shown in Figure 6a–c, with values listed in Table 2). The relationship between charge and capacitance is described in classical electromagnetism theory [32]. Using the formula

(2) $$\Delta Q=C_{barrier}\times \Delta V_{th}$$

where the barrier capacitance was measured to be $3.18\times 10^{-7}F/{cm}^{2}$, the charge density can be calculated. Since CPT is not charged (Figure 6a, $∆V_{th}=0\;V$), the change in $I_{ds}$ in the gate area must be attributed to the effect of CPT on the cells. The charge density change in RAJI cell fluid due to CPT is $2.89\times 10^{11}/{cm}^{2}$, whereas the charge density change in WIL2S cell fluid caused by CPT is not significant (Figure 6b, $∆V_{th}=0.09\;V$, with an error of 0.09 V for $V_{th}$).

The effects of DOX on both cell lines (Figure 6c) are weaker compared to those of CPT (Figure 6b). From the time constant τ (Table 1), it is observed that the interaction processes of the two drugs with normal B cells (WIL2S) are faster than with RAJI cells (cancerous cell) (see τ in Table 1). For the same cell line (RAJI or WIL2S), CPT treatment involves two processes: an initial rapid response followed by a slower process, whereas DOX displays only single phase during the measurement period. The fluorescence measurements of drug molecules in the cells at different times reveal that CPT enters cells very rapidly, while DOX enters the same type of cells relatively slowly.

### 3.4. The Speed of the Two Drugs on Cells Confirmed by Flow Cytometry

**Figure 7:**
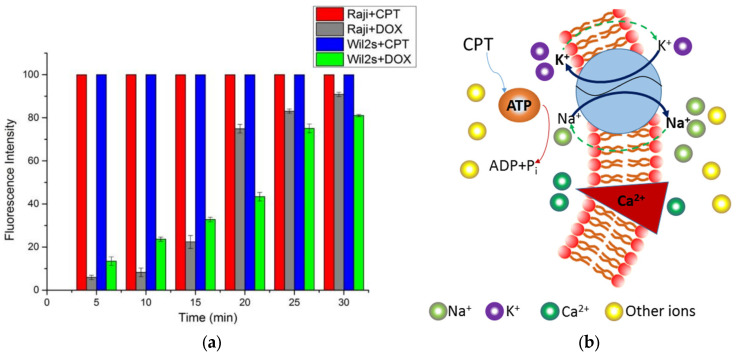
Drug uptake over time and proposed mechanism of CPT action on RAJI Cells. (**a**) The result of drug uptake into cells at various times, as determined by flow cytometry. (**b**) A possible mechanism by which CPT acts on RAJI cells.

Flow cytometry was used to verify the speed at which the two drugs act on the cells (see Figure 7a for the results). The histogram reveals that CPT rapidly entered both cell types. In contrast, DOX entered WIL2S cells faster than RAJI cells within the first 15 min, which aligns with the time constants obtained from the electrical measurements. Following this initial period, DOX entered WIL2S cells at a relatively slower rate compared to the earlier stage of entry.

## 4. Discussion

In this study, the exposure of RAJI cells to CPT resulted in a significant increase in $I_{ds}$. We hypothesize that CPT treatment indirectly stimulates ATP hydrolysis and activates ion pumps, leading to an increase in positive ions or a decrease in negative ions in the solution [33] (see Figure 7b). This, in turn, raises the total number of positive ions outside the cells, thereby increasing $I_{ds}$ (Figure 5a).

This hypothesis may be supported by findings from two additional studies: one on the effects of CPT treatment on single U251 cells [34] and another on thermogenesis in living cells [24]. The study on U251 cells observed an acute increase in intracellular temperature following CPT treatment. Similarly, the addition of ionomycin calcium complexes to single NIH/3T3 cells resulted in elevated intracellular Ca^2+^ concentrations and a noticeable temperature rise within the cell. In contrast, the effect of CPT and DOX on noncancerous cell lines appears to induce only minimal ion pump activity, leading to negligible changes in ion concentration and a relatively modest temperature rise in single cells [34].

For DOX, we suspect that very little of the drug enters the cell within the given time frame [35], a hypothesis supported by the results shown in Table 1 and Figure 7a. The overall results indicate a slight decrease in the concentration of positive ions in the gate area, but not to a significant extent.

For B lymphocytes (WIL2S), the effects of the anti-cancer drugs (CPT and DOX) were less pronounced compared to cancerous RAJI cells. The net changes in $I_{ds}$ are illustrated as follows: Figure 5b shows an increase of approximately 1.5 µA for CPT treatment on RAJI cells, Figure 5c shows an increase of approximately 0.2 µA for CPT treatment on WIL2S cells, Figure 5e shows a change of around 0.2 μA for DOX treatment to RAJI cells, and Figure 5f shows a change of 0.1 µA for DOX treatment on WIL2S cells. These results demonstrate the anti-tumor specificity of the two drugs to cancerous cells to some extent.

Based on both current–voltage measurements (Figure 5) and the evaluation of time constants in each case (Table 1), we conclude that only CPT significantly affects the cancerous (RAJI) cell line within the observed time frame, while WILS2 cells are relatively unaffected by either CPT or DOX. Given the slower action process of DOX, it is likely that DOX does interact with ion pumps within RAJI cells, but these effects are not detectable within the short duration of our experiments.

However, this study also has the following limitations: First, the range of drug selection is limited, as only two typical anti-cancer drugs (camptothecin and doxorubicin) were investigated. This may restrict the representativeness of the findings. Future research will consider expanding the range of drugs, including those with different mechanisms of action, to validate the broad applicability of the method. Additionally, the experiment used only one cancer cell line (RAJI) and one non-cancerous cell line (WIL2S), which may not sufficiently represent the responses of various cell types to drugs. We plan to include multiple cancer and normal cell lines in future studies, particularly those derived from different tissues or with distinct characteristics. Furthermore, the study primarily focused on the short-term effects of drugs, without addressing their long-term impacts on cells, such as apoptosis rates or drug resistance. Subsequent research will investigate the long-term effects of drugs to provide a more comprehensive understanding of their impact on cells.

## 5. Conclusions

In conclusion, this study presents a viable physical approach for the rapid and straightforward assessment of drug effects on both cancerous and normal cells, focusing on action speed and charge change using AlGaN/GaN field-effect transistors over a short timeframe. The results demonstrate that CPT has a significant impact on RAJI cells, leading to an increase in ion concentration in the solution. In contrast, the effects of DOX, another common chemotherapy drug, are less pronounced within the same short time period. Additionally, the study reveals that both drugs have a more pronounced effect on cancerous cells within the short experimental duration. This approach offers a new method for drug screening in cancer treatment, initial efficacy evaluation, and the research and development of new drugs. By distinguishing between the effects on cancerous and normal cells, this technique supports the development of targeted and more effective cancer treatments. It also aids in understanding drug action mechanisms to some extent, potentially contributing to improved therapeutic effect evaluations and disease treatment strategies.

## Figures and Tables

**Table 1:** Response time constant τ for various conditions corresponding to Figure 5a–f.

Drug	RAJI Cell	1640	WIL2S Cell
CPT	$\tau_{1}=23.03\;s$ $\tau_{2}=393.54\;s$	none	$\tau_{1}=7.46\;s$ $\tau_{2}=309.15\;s$
DOX	τ=28.00 s	τ=48.37 s	τ=25.85 s

**Table 2:** The threshold voltage ($V_{th}$) of the gate region under different conditions corresponding to Figure 6a,c.

Drug	RAJI Cell	1640	WIL2S Cell
CPT	−4.19 V	−4.65 V	−4.10 V
DOX	−4.10 V	−4.06 V	−4.11 V

## Data Availability

Data are contained within the article.

## References

[1] Liu B., Zhou H., Tan L., Siu K.T.H., Guan X.Y. (2024). Exploring treatment options in cancer: Tumor treatment strategies. Signal Transduct. Target. Ther..

[2] Petrowsky H., Fritsch R., Guckenberger M., De Oliveira M.L., Dutkowski P., Clavien P.A. (2020). Modern therapeutic approaches for the treatment of malignant liver tumours. Nat. Rev. Gastroenterol. Hepatol..

[3] Passaro A., Al Bakir M., Hamilton E.G., Diehn M., André F., Roy-Chowdhuri S., Mountzios G., Wistuba I.I., Swanton C., Peters S. (2024). Cancer biomarkers: Emerging trends and clinical implications for personalized treatment. Cell.

[4] Niu H., Li K.Y., Yu T., Zhang M., Ji Z., Yu P., Yi X., Liu G. (2024). Worldwide Research Trends and Regional Differences in the Development of Precision Medicine Under Data-Driven Approach: A Bibliometric Analysis. J. Multidiscip. Health.

[5] Duan X.P., Qin B.D., Jiao X.D., Liu K., Wang Z., Zang Y.S. (2024). New clinical trial design in precision medicine: Discovery, development and direction. Signal Transduct. Target. Ther..

[6] Zhang Z., Zhou L., Xie N., Nice C.E., Zhang T., Cui Y.P., Huang C. (2020). Overcoming cancer therapeutic bottleneck by drug repurposing. Signal Transduct. Target. Ther..

[7] Li W., Yang J., Luo L., Jiang M., Qin B., Yin H., Zhu C., Yuan X., Zhang J., Luo Z. (2019). Targeting photodynamic and photothermal therapy to the endoplasmic reticulum enhances immunogenic cancer cell death. Nat. Commun..

[8] Dallavalle S., Dobričić V., Lazzarato L., Gazzano E., Machuqueiro M., Pajeva I., Tsakovska I., Zidar N., Fruttero R. (2020). Improvement of conventional anti-cancer drugs as new tools against multidrug resistant tumors. Drug Resist. Updates.

[9] Dolphin A.C., Lee A. (2020). Presynaptic calcium channels: Specialized control of synaptic neurotransmitter release. Nat. Rev. Neurosci..

[10] Bunea A.I., Harloff-Helleberg S., Taboryski R., Nielsen H.M. (2020). Membrane interactions in drug delivery: Model cell membranes and orthogonal techniques. Adv. Colloid Interface Sci..

[11] Yang B., Yang H., Liang J., Chen J., Wang C., Wang Y., Wang J., Luo W., Deng T., Guo J. (2024). A review on the screening methods for the discovery of natural antimicrobial peptides. J. Pharm. Anal..

[12] Ashrafuzzaman M., Khan Z., Alqarni A., Alanazi M., Alam M.S. (2021). Cell surface binding and lipid interactions behind chemotherapy-drug-induced ion pore formation in membranes. Membranes.

[13] Mosgaard L.D., Heimburg T. (2013). Lipid ion channels and the role of proteins. Acc. Chem. Res..

[14] Ghatak S., Diedrich J.K., Talantova M., Bhadra N., Scott H., Sharma M., Albertolle M., Schork N.J., Yates J.R., Lipton S.A. (2024). Single-Cell Patch-Clamp/Proteomics of Human Alzheimer’s Disease iPSC-Derived Excitatory Neurons Versus Isogenic Wild-Type Controls Suggests Novel Causation and Therapeutic Targets. Adv. Sci..

[15] Spira M.E., Hai A. (2013). Multi-electrode array technologies for neuroscience and cardiology. Nat. Nanotechnol..

[16] Ju Y., Li H., Li J., Gu N., Yang F. (2023). Dual-parameter cell biosensor for real-time monitoring of effects of propionic acid on neurons. Biosens. Bioelectron..

[17] Wang S., Zhang J., Gharbi O., Vivier V., Gao M., Orazem M.E. (2021). Electrochemical impedance spectroscopy. Nat. Rev. Methods Primers.

[18] Fiala T., Wang J., Dunn M., Šebej P., Choi S.J., Nwadibia E.C., Fialova E., Martinez D.M., Cheetham C.E., Fogle K.J. (2020). Chemical targeting of voltage sensitive dyes to specific cells and molecules in the brain. J. Am. Chem. Soc..

[19] Klier P.E., Gest A.M., Martin J.G., Roo R., Navarro M.X., Lesiak L., Deal P.E., Dadina N., Tyson J., Schepartz A. (2022). Bioorthogonal, fluorogenic targeting of voltage-sensitive fluorophores for visualizing membrane potential dynamics in cellular organelles. J. Am. Chem. Soc..

[20] Emiliani V., Entcheva E., Hedrich R., Hegemann P., Konrad K.R., Lüscher C., Mahn M., Pan Z.-H., Sims R.R., Vierock J. (2022). Optogenetics for light control of biological systems. Nat. Rev. Methods Primers.

[21] Lu W., Kumar V., Schwindt R., Piner E., Adesida I. (2002). A comparative study of surface passivation on AlGaN/GaN HEMTs. Solid-State Electron..

[22] Wen X., Schuette M.L., Gupta S.K., Nicholson T.R., Lee S.C., Lu W. (2010). Improved sensitivity of AlGaN/GaN field effect transistor biosensors by optimized surface functionalization. IEEE Sens. J..

[23] Vashishtha P., Abidi I.H., Giridhar S.P., Verma A.K., Prajapat P., Bhoriya A., Murdoch B.J., Tollerud J.O., Xu C., Davis J.A. (2024). CVD-Grown Monolayer MoS2 and GaN Thin Film Heterostructure for a Self-Powered and Bidirectional Photodetector with an Extended Active Spectrum. ACS Appl. Mater. Interfaces.

[24] Wang Y., Lu W. (2011). AlGaN/GaN FET for DNA hybridization detection. Phys. Status Solidi (a).

[25] Gupta S., Elias M., Wen X., Shapiro J., Brillson L., Lu W., Lee S.C. (2008). Detection of clinically relevant levels of protein analyte under physiologic buffer using planar field effect transistors. Biosens. Bioelectron..

[26] Mishra S., Kachhawa P., Jain A.K., Thakur R.R., Chaturvedi N. (2022). High sensitivity label-free detection of HER2 using an Al–GaN/GaN high electron mobility transistor-based biosensor. Lab Chip.

[27] Upadhyay K.T., Chattopadhyay M.K. (2021). Sensor applications based on AlGaN/GaN heterostructures. Mater. Sci. Eng. B.

[28] Yuan H., Jiang A., Fang H., Chen Y., Guo Z. (2021). Optical properties of natural small molecules and their applications in imaging and nanomedicine. Adv. Drug Deliv. Rev..

[29] González-Ruiz V., Cores Á., Martín-Cámara O., Orellana K., Cervera-Carrascón V., Michalska P., Olives A.I., León R., Martín M.A., Menéndez J.C. (2021). Enhanced stability and bioactivity of natural anticancer topoisomerase i inhibitors through cyclodextrin complexation. Pharmaceutics.

[30] Zhang M., Chen X., Li C., Shen X. (2020). Charge-reversal nanocarriers: An emerging paradigm for smart cancer nanomedicine. J. Control. Release.

[31] Lingeshwar Reddy K., Balaji R., Kumar A., Krishnan V. (2018). Lanthanide doped near infrared active upconversion nanophosphors: Fundamental concepts, synthesis strategies, and technological applications. Small.

[32] Das S. (2019). Theoretical verification of formula for charge function in time q = c* v in RC circuit for charging/discharging of fractional & ideal capacitor. Asian J. Res. Rev. Phys..

[33] Godoy-Gallardo M., Eckhard U., Delgado L.M., de Roo Puente Y.J., Hoyos-Nogués M., Gil F.J., Perez R.A. (2021). Antibacterial approaches in tissue engineering using metal ions and nanoparticles: From mechanisms to applications. Bioact. Mater..

[34] Wang C., Xu R., Tian W., Jiang X., Cui Z., Wang M., Sun H., Fang K., Gu N. (2011). Determining intracellular temperature at single-cell level by a novel thermocouple method. Cell Res..

[35] L-Kareh A.W., Secomb T.W. (2005). Two-mechanism peak concentration model for cellular pharmacodynamics of Doxorubicin. Neoplasia.

